# Cognition at age 70: Life course predictors and associations with brain pathologies

**DOI:** 10.1212/WNL.0000000000008534

**Published:** 2019-12-03

**Authors:** Kirsty Lu, Jennifer M. Nicholas, Jessica D. Collins, Sarah-Naomi James, Thomas D. Parker, Christopher A. Lane, Ashvini Keshavan, Sarah E. Keuss, Sarah M. Buchanan, Heidi Murray-Smith, David M. Cash, Carole H. Sudre, Ian B. Malone, William Coath, Andrew Wong, Susie M.D. Henley, Sebastian J. Crutch, Nick C. Fox, Marcus Richards, Jonathan M. Schott

**Affiliations:** From the Dementia Research Centre (K.L., J.D.C., T.D.P., C.A.L., A.K., S.E.K., S.M.B., H.M.-S., D.M.C., C.H.S., I.B.M., W.C., S.M.D.H., S.J.C., N.C.F., J.M.S.), UCL Queen Square Institute of Neurology, University College London; Department of Medical Statistics (J.M.N.), London School of Hygiene and Tropical Medicine; MRC Unit for Lifelong Health and Ageing at UCL (S.-N.J., A.W., M.R.); and School of Biomedical Engineering and Imaging Sciences (D.M.C., C.H.S.), King's College London, UK.

## Abstract

**Objective:**

To investigate predictors of performance on a range of cognitive measures including the Preclinical Alzheimer Cognitive Composite (PACC) and test for associations between cognition and dementia biomarkers in Insight 46, a substudy of the Medical Research Council National Survey of Health and Development.

**Methods:**

A total of 502 individuals born in the same week in 1946 underwent cognitive assessment at age 69–71 years, including an adapted version of the PACC and a test of nonverbal reasoning. Performance was characterized with respect to sex, childhood cognitive ability, education, and socioeconomic position (SEP). In a subsample of 406 cognitively normal participants, associations were investigated between cognition and β-amyloid (Aβ) positivity (determined from Aβ-PET imaging), whole brain volumes, white matter hyperintensity volumes (WMHV), and *APOE ε4*.

**Results:**

Childhood cognitive ability was strongly associated with cognitive scores including the PACC more than 60 years later, and there were independent effects of education and SEP. Sex differences were observed on every PACC subtest. In cognitively normal participants, Aβ positivity and WMHV were independently associated with lower PACC scores, and Aβ positivity was associated with poorer nonverbal reasoning. Aβ positivity and WMHV were not associated with sex, childhood cognitive ability, education, or SEP. Normative data for 339 cognitively normal Aβ-negative participants are provided.

**Conclusions:**

This study adds to emerging evidence that subtle cognitive differences associated with Aβ deposition are detectable in older adults, at an age when dementia prevalence is very low. The independent associations of childhood cognitive ability, education, and SEP with cognitive performance at age 70 have implications for interpretation of cognitive data in later life.

Alzheimer disease (AD) has a preclinical window extending perhaps 20 years before the onset of symptoms^1^ and research criteria are increasingly using biomarkers to identify individuals with preclinical disease.^2,3^ Accumulation of brain β-amyloid (Aβ) is a very early feature of the disease process.^4^ There is therefore considerable interest in identifying individuals who are Aβ-positive (Aβ+) for recruitment to preclinical AD trials.

Emerging evidence suggests that subtle cognitive decline is present in this preclinical phase,^5^ which has led to efforts to develop sensitive cognitive measures such as the Preclinical Alzheimer Cognitive Composite (PACC)^6^ to detect and track this decline. Cognitively normal Aβ+ older adults have shown faster decline on the PACC than Aβ− individuals,^7,8^ but evidence for cross-sectional differences on the PACC between Aβ groups is mixed.^6–12^

The life course determinants of performance on the PACC in older age are unknown. Insight 46, a substudy of the National Survey of Health and Development (NSHD) (British 1946 Birth Cohort), is uniquely placed to address this, since data are available on participants’ cognition since childhood. Participants were assessed at an age where the prevalence of dementia is low (∼3%),^13^ but 15%–25% are expected to be Aβ+.^14^

This study aimed first to characterize the performance of Insight 46 participants on cognitive tests including the PACC with respect to sex, childhood cognitive ability, education, and adult socioeconomic position. We then explored whether cognitive performance was influenced by amyloid status, structural MRI biomarkers including brain and white matter hyperintensity volumes, and genetic risk for AD (*APOE* ε4).

## Methods

**Figure 1:**
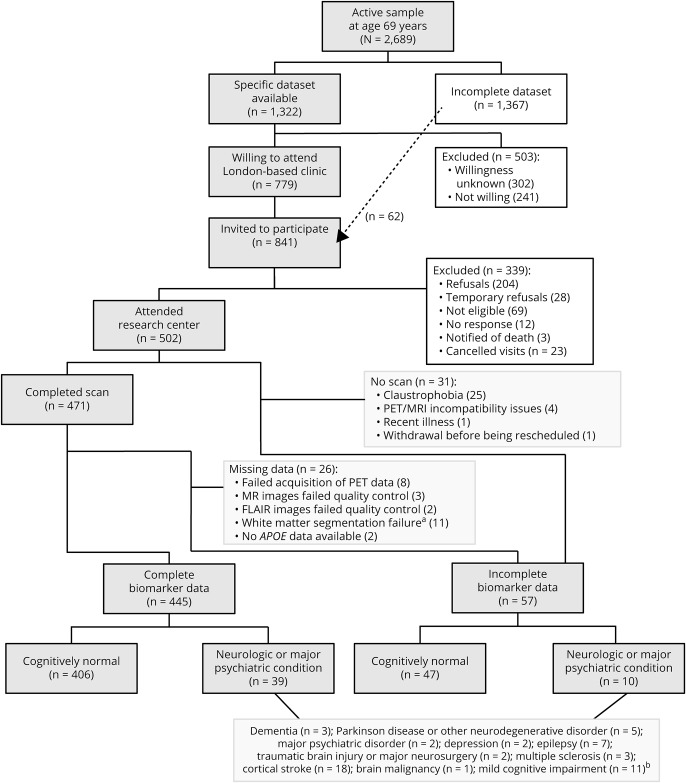
Flowchart of recruitment and data acquisition The specific dataset refers to a set of life course data that formed the original criteria for Insight 46 eligibility. See reference 17 for further details. To reach our target sample size, these criteria were relaxed to remove the requirement for a previous measure of lung function, smoking, or physical exercise. ^a^ In most cases, this was due to erroneous segmentation of vascular abnormalities such as stroke or demyelination. ^b^ These numbers add up to 54 because some participants had more than one condition. See Methods for details of the definitions of neurologic and psychiatric disorders. FLAIR = fluid-attenuated inversion recovery.

The NSHD is a population-based cohort of 5,362 men and women born across mainland Britain during 1 week in March 1946. With 24 data collections across childhood and adulthood, most recently at age 68–69, it is the world's longest continuously running birth cohort.^15^ For the Insight 46 neuroscience substudy, 502 NSHD participants were recruited and assessed at a clinic in University College London between May 2015 and January 2018. Recruitment procedures have been described previously^16,17^ and are summarized in figure 1. Measures included cognitive tests, clinical history and examination, Aβ-PET imaging, brain MRI, and other biomarker and genetic measures as detailed elsewhere.^16^ Each participant had an informant who completed the AD8 interview, a brief screening tool for dementia.^18^

### Standard protocol approvals, registrations, and patient consents

The study was approved by the National Research Ethics Service Committee London (REC reference 14/LO/1173) and all participants provided written informed consent.

### Cognitive assessment

The original PACC is composed of 4 cognitive tests: the Mini Mental State Examination (MMSE), Logical Memory IIa from the Wechsler Memory Scale-Revised, Digit-Symbol Substitution test (DSST) from the Wechsler Adult Intelligence Scale-Revised, and the Free and Cued Selective Reminding Test (FCSRT).^6^ Several variations of the PACC have been tested.^6–8,19–22^ We replaced the FCSRT with the 12-item Face-Name test (FNAME-12),^23^ to avoid potential overlap with a similar word-learning memory test administered to the NSHD cohort at multiple time-points throughout adulthood.^24^ FNAME-12 is similar to FCSRT in terms of being an episodic memory test of immediate and delayed recall, is moderately correlated with FCSRT free recall scores^23^ and is also relatively challenging for cognitively normal populations. Two previous studies have reported that FNAME is sensitive to Aβ deposition.^25,26^

Participants also completed the Matrix Reasoning test from the Wechsler Abbreviated Scale of Intelligence^27^—a measure that was chosen for its similarity to aspects of the cognitive tests completed in childhood.

The MMSE^28^ is a 30-point composite screening tool for cognitive impairment that is widely used within clinical practice.

The DSST^29^ is an index of executive function and psychomotor speed. The score is the number of items completed correctly within 90 seconds.

Logical Memory IIa^30^ assesses free recall of a short story, which the participant is asked to recall immediately and after a delay of approximately 20 minutes.

The FNAME-12^23^ assesses associative memory for face–name and face–occupation pairs. Two versions exist: FNAME-12A and FNAME-12B. This study used FNAME-12A. Participants are shown 12 unfamiliar face–name and face–occupation pairs (e.g., “Sarah, reporter”), with 8 seconds to study each one. They are then presented with each face and asked to recall the associated name and occupation. This process is repeated with a second learning phase and a second recall test. After a ∼10-minute delay, they are again shown each face and asked to recall the names and occupations (the third recall test). After a ∼30-minute delay, participants are shown 12 sets of 3 faces and asked to identify each previously learned face from the 2 distractors (facial recognition) and to recall the name and occupation (the fourth recall test). If they cannot recall the name or occupation, they are asked to select the correct answer from 3 options comprising the correct answer, a distractor (a name/occupation that belongs with a different face in the set), and a name/occupation that did not feature in the set. The summary outcomes are FN-N (total names recalled, maximum 48), FN-O (total occupations recalled, maximum 48), and FNAME-total (FN-N + FN-O, maximum 96)—these outcomes are based on the 4 recall tests. Precise administration times were recorded for a sample of 50 participants to check that the delay times conformed to expectations: the mean delay times were 10.0 minutes and 35.5 minutes.

The Matrix Reasoning Test assesses nonverbal reasoning, an aspect of fluid intelligence. Participants are shown a matrix of geometric shapes and are required to select the missing piece from 5 options. There are 32 matrices, graded in difficulty, and the test is discontinued when participants make 4 consecutive errors (or 4 errors within 5 consecutive items), as specified in the manual.^27^

The 4 components of our version of the PACC were MMSE total score, Logical Memory delayed recall score, DSST score, and FNAME-total. Following the method described in previous studies,^8,11,12,19,20^ the 4 components were converted into *z* scores based on the full Insight 46 sample, and then averaged. A higher PACC score indicates better performance. Two participants did not complete the FNAME test and one did not complete the DSST. For these 3 participants, their PACC score was the average of the *z* scores for the 3 tests they completed. This is consistent with a previous study that required at least 2 out of the 4 components to be present.^10^ Excluding these 3 people did not change any of the results.

### Life course and clinical variables

Childhood cognitive ability was measured at age 8 using 4 tests of verbal and nonverbal ability devised by the National Foundation for Education Research.^31^ The sum of scores from these 4 tests was standardized into a *z* score representing overall cognitive ability. If these data were missing, the equivalent score from the tests at age 11 was used (or if this was missing, the score from age 15 was used). These standardized scores were based on the full NSHD cohort.

Educational attainment was represented as the highest educational or training qualification achieved by age 26, grouped into 5 categories: no qualification, below O-levels (vocational), O-levels and equivalents, A-levels and equivalents, higher education (degree and equivalents).

Adult socioeconomic position (SEP) was derived from participants’ own occupation at age 53, or earlier if this was missing. Occupations were coded according to the UK Registrar General's Standard Occupational Classification, then classified into 6 categories: unskilled, partly skilled, skilled manual, skilled nonmanual, intermediate, professional.

Participants were coded as having a neurologic or major psychiatric condition if they met any of the following criteria: (1) clinical evidence of dementia, Parkinson disease, or other neurodegenerative disorder; (2) psychiatric disorder requiring antipsychotic medication; (3) depression requiring electroconvulsive shock therapy; (4) epilepsy requiring active treatment; (5) radiologic evidence of traumatic brain injury or major neurosurgery; (6) clinical diagnosis or radiologic features of multiple sclerosis; (7) clinical diagnosis of stroke, or radiologic evidence of cortical ischemia or hemorrhage consistent with previous cortical stroke; (8) radiologic evidence of possible brain malignancy; (9) mild cognitive impairment (MCI) defined as follows, based on published criteria^32^:

1. no clinical evidence of dementia; and
2. participant concern regarding cognition (memory or cognitive difficulties more than other people the same age, or if they reported that they would seek medical attention regarding their difficulties) or informant concern regarding the participant's cognition (AD8 score ≥2); and
3. objective evidence of either an amnestic (Logical Memory delayed recall ≥1.5 SD below the mean) or nonamnestic deficit (DSST score ≥1.5 SD below the mean). These cognitive tests were chosen for defining a cognitive deficit on the basis of their normal distribution across the entire sample.

Participants not meeting any of these criteria are hereafter referred to as cognitively normal and represent a sample who might be considered eligible for a clinical trial of cognitively healthy individuals, free from possible confounding comorbidities. This does not imply that all participants with a neurologic or major psychiatric condition necessarily had a measurable cognitive impairment. Numbers of participants in each category are detailed in figure 1.

### Biomarker measures

Aβ-PET and multimodal MRI data were collected simultaneously during a 60-minute scanning session on a single Biograph mMR 3T PET/MRI scanner (Siemens Healthcare, Erlangen, Germany), with IV injection of 370 MBq of the Aβ-PET ligand, ^18^F-Florbetapir (Amyvid). Protocol details have been described elsewhere.^16^

Aβ deposition was quantified using a standard uptake volume ratio (SUVR), calculated from cortical regions of interest with a reference region of eroded subcortical white matter, using 10 minutes of static steady-state florbetapir data ∼50 minutes postinjection. A cutpoint for Aβ positivity was determined using a mixture model to define 2 Gaussians, and using the 99th percentile of the lower (Aβ negative) Gaussian, at SUVR >0.6104. Aβ-PET attenuation correction was performed using pseudo-CT correction.^16^ Due to technical issues, only console attenuation correction was available for 26 participants. For these participants, a pseudo-CT corrected value was imputed based on the console value.

Whole brain volume was generated from high-resolution 3D T1-weighted MRI using automated segmentation with manual editing.^16^ Total intracranial volume (TIV) was calculated using statistical parametric mapping (SPM) software (SPM12; fil.ion.ucl.ac.uk/spm).^33^ Global white matter hyperintensity volume (WMHV) was generated from multimodal MRI using an automated segmentation algorithm based on a multivariate Gaussian mixture model,^34^ followed by visual quality control, generating a global WMHV including subcortical gray matter but excluding infratentorial regions.

*APOE* genotype was determined from DNA analysis of blood samples as previously described^16^ and classified into 3 categories based on the presence of the ε4 allele: no ε4, ε4 heterozygous, and ε4 homozygous.

Fifty-seven participants were missing biomarker data (figure 1).

### Data analyses

To investigate the relationship between cognitive outcomes and demographic factors we included all participants (n = 502), as we aimed to describe the predictors of cognitive performance in as representative a sample as possible. Raw scores from each cognitive test were standardized to *z* scores based on the full Insight 46 sample to allow comparison of effect sizes across different cognitive tests. Multivariable linear regression models were run where the outcome was the *z* score on a particular cognitive test and the predictors were sex, age at assessment, childhood cognitive ability, education, adult SEP, and presence of a neurologic or major psychiatric condition (including MCI). Examination of residuals was performed to check model fits. For outcomes with skewed distributions (MMSE and Matrix Reasoning), bootstrapping was used to produce bias-corrected and accelerated 95% confidence intervals (CIs) from 2,000 replications. For the Logical Memory delayed recall score, the model contained an additional factor of delay duration (time elapsed between the immediate and delayed recall). Mean delay duration was 24.6 minutes (SD 4.66) and there was no evidence that this was associated with performance (regression coefficient = −0.006, 95% CIs −0.024 to 0.012, *p* = 0.53), but it was included in the models as per standard practice.

To investigate associations between cognitive performance and biomarkers of brain pathology, we included only those participants classified as cognitively normal (i.e., no dementia, non-MCI population free from possible confounding comorbidities), and for whom all biomarker data were available (n = 406). The *z* score on a particular cognitive test was the outcome and Aβ status, whole brain volume, WMHV, and *APOE* genotype were included as predictors in multivariable regression models to examine the effects of each biomarker adjusted for all the others. To adjust for the correlation between whole brain volume and head size, TIV was included in all models, as were the demographic and life course factors investigated in the first analysis (sex, age at assessment, childhood cognitive ability, education, and adult SEP). Interactions were investigated between amyloid status and brain volumes, and amyloid status and WMHV.

The models were in addition rerun replacing dichotomized amyloid status with a continuous measure of Aβ (SUVR) to test whether increasing Aβ deposition was associated with differences in performance. To check whether associations between SUVR and cognition were sensitive to the inclusion of the imputed SUVR values, the analyses were rerun excluding the 26 participants with imputed data.

All analyses were conducted using Stata 15 (StataCorp, College Station, TX). Statistical significance was set at *p* < 0.05.

### Data availability

Anonymized data will be shared by request from qualified investigators (skylark.ucl.ac.uk/NSHD/doku.php).

## Results

Participant characteristics are reported in table 1. Descriptive statistics for each test are given in table 2. Normative data for cognitively normal Aβ− participants are available in table e-1 (doi.org/10.5061/dryad.nt561v7).

**Table 1:**
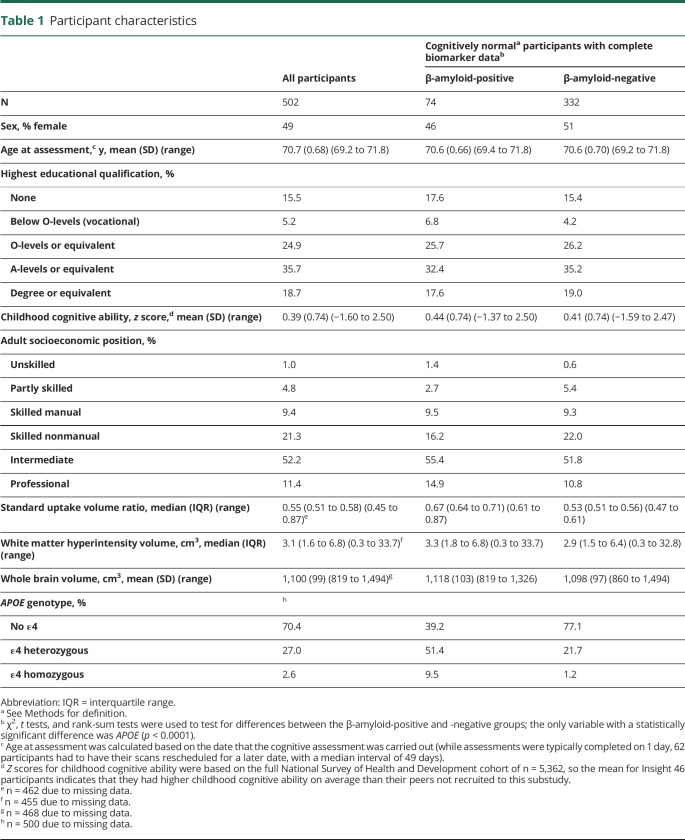
Participant characteristics

**Table 2:**
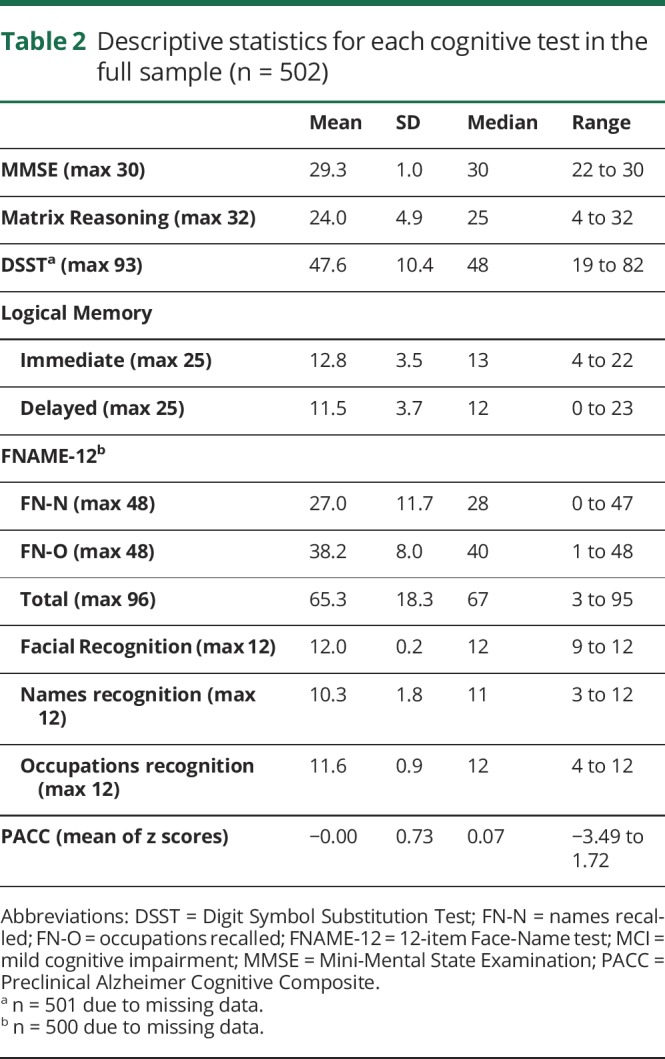
Descriptive statistics for each cognitive test in the full sample (n = 502)

On average, Insight 46 participants performed at the expected level for their age on the MMSE, DSST, and Logical Memory tests, according to normative data.^35^ On the Matrix Reasoning test, their performance (mean 24) was above the expected level based on normative data (sample mean for 70- to 74-year-olds is 16)^27^ but comparable to a sample of healthy older adults recruited by Washington University (mean 24).^36^ To date, only 2 studies have published FNAME-12 data^23,37^ and Insight 46 means are higher than these.

### Predictors of performance

Results of the multivariable regression models exploring associations with demographic and life course predictors are reported in table 3. On average, participants with neurologic or major psychiatric conditions (including MCI) scored significantly lower on all tests (table 3). The analyses were rerun excluding the participants with MCI to check that these differences could not be explained by circularity in the definition of MCI (since low scores on the Logical Memory or DSST formed part of the MCI criteria); the results were unchanged except that the differences were no longer statistically significant on MMSE and Matrix Reasoning.

**Table 3:**
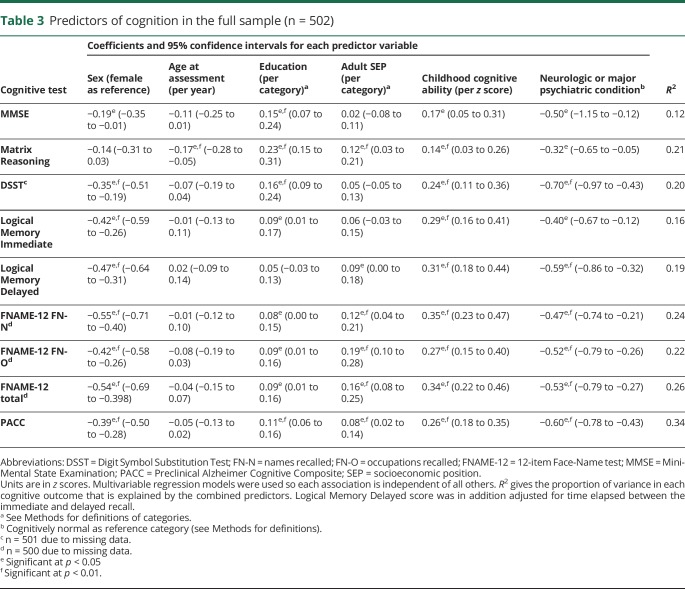
Predictors of cognition in the full sample (n = 502)

Female participants scored significantly higher than male participants on all measures except Matrix Reasoning (table 3); the greatest difference was on the FNAME-12, particularly in recalling names.

As expected across this narrow age range (2.6 years; reflecting the time it took to collect the data, since participants were all born in the same week), there was no evidence of age effects on cognition, except on the Matrix Reasoning test, where older age was associated with slightly poorer performance (table 3).

**Figure 2:**
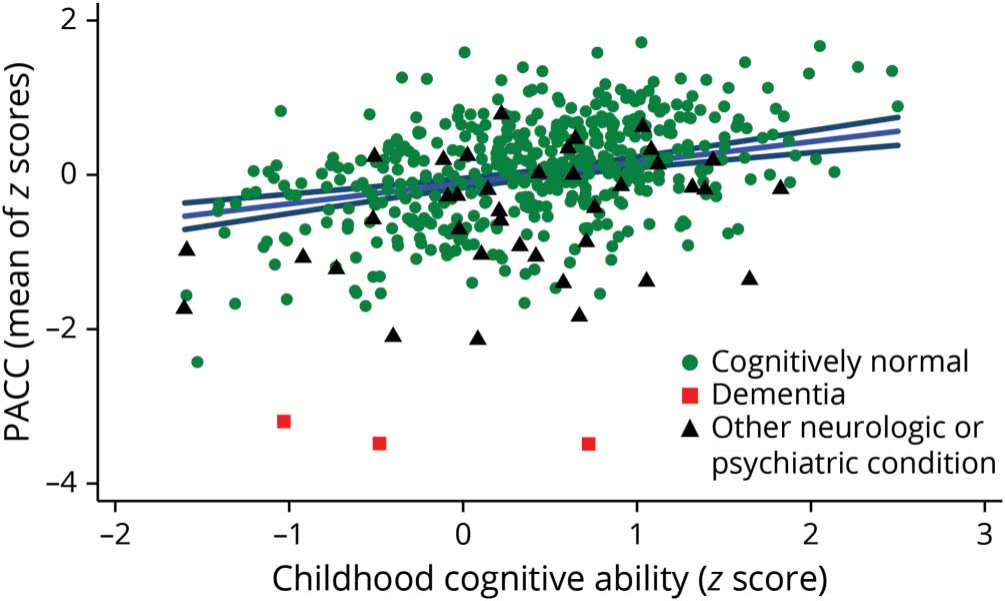
Preclinical Alzheimer Cognitive Composite (PACC) score against childhood cognitive ability Scatterplot shows the raw data, color-coded by clinical group. Alzheimer disease dementia is distinguished from other neurologic or major psychiatric conditions for interest. The blue line is the line of best fit from the multivariable regression model (adjusted for sex, age at assessment, education, adult socioeconomic position, and presence of neurologic or major psychiatric conditions), and the navy lines are its 95% confidence intervals. *Z* scores for childhood cognitive ability were based on the full National Survey of Health and Development cohort (n = 5,362).

Higher childhood cognitive ability was associated with better performance on every cognitive outcome (table 3 and figure 2). Higher educational attainment and higher adult SEP were independently positively associated with the majority of cognitive outcome measures, including the PACC. Higher educational attainment showed a notable positive association with the Matrix Reasoning task.

All these effects were maintained when excluding participants with neurologic or major psychiatric conditions, except that the following 2 associations were directionally but no longer statistically significant: Logical Memory Delayed and SEP (*p* = 0.073); FNAME FN-O and education (*p* = 0.12).

### Associations with biomarkers

Results of the multivariable regression models are reported in table 4.

**Table 4:**
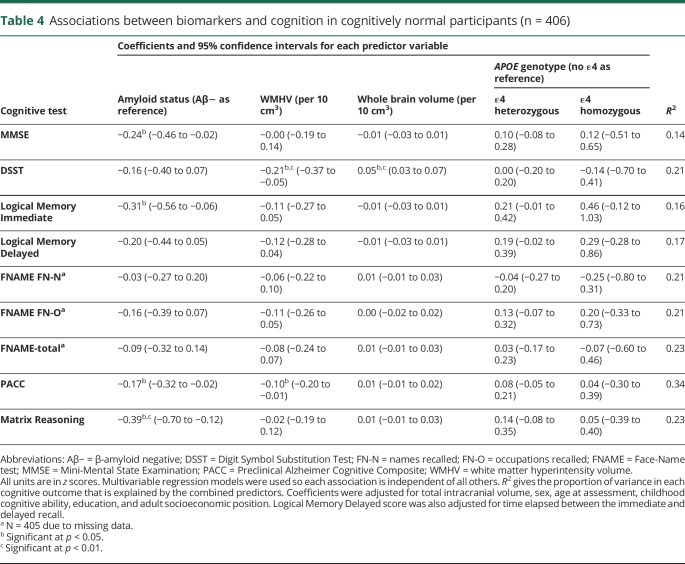
Associations between biomarkers and cognition in cognitively normal participants (n = 406)

Of the cognitively normal participants with complete biomarker data, 18.3% were classified as Aβ+ (table 1), which is around the expected prevalence for this age.^14^ There were no statistically significant differences between the Aβ+ and Aβ− groups by sex, age at assessment, childhood cognitive ability, education, adult SEP, brain volume, or WMHV (table 1). As expected, the Aβ+ group contained a higher proportion of *APOE* ε4 carriers (table 1). There were no statistically significant associations between WMHV and sex, childhood cognitive ability, education, SEP, brain volume, or *APOE* genotype, but there was a weak association between older age and greater WMHV (Spearman ρ = 0.12, *p* = 0.016).

**Figure 3:**
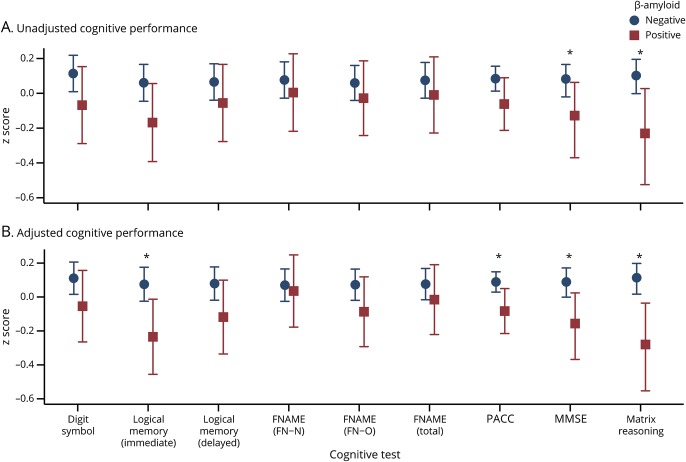
Cognitive performance for β-amyloid-positive and β-amyloid-negative individuals: means and 95% confidence intervals (A) Unadjusted means. (B) Adjusted means predicted from the multivariable regression models (adjusted for age at assessment, sex, childhood cognitive ability, education, adult socioeconomic position, whole brain volume, total intracranial volume, white matter hyperintensity volume, and *APOE* genotype). Asterisks indicate statistically significant differences (*p* < 0.05). FN-N = total names recalled; FN-O = total occupations recalled; FNAME-12 = 12-item Face-Name test; MMSE = Mini-Mental State Examination; PACC = Preclinical Alzheimer Cognitive Composite.

On average, Aβ+ participants scored lower than Aβ− participants on every cognitive measure (table 4 and figure 3). The unadjusted differences were only statistically significant for the MMSE and Matrix Reasoning (figure 3A), but in the multivariable model adjusting for demographic, life course, and biomarker factors, the differences were also statistically significant for Logical Memory immediate recall and PACC (figure 3B and table 4).

Replacing dichotomized amyloid status with the continuous SUVR revealed weak associations between higher SUVR and poorer performance on MMSE (regression coefficient -1.21, 95% CIs −2.39 to −0.10), Logical Memory immediate recall (regression coefficient −1.87, 95% CIs −3.22 to −0.52), and PACC (regression coefficient −1.09, 95% CIs −1.90 to −0.29). Similar trends were observed on the other tests but did not reach statistical significance. These results were unchanged when the analyses were rerun excluding the 26 participants with imputed SUVR data.

The only outcome that showed an association with whole brain volume was DSST, where larger whole brain volume was associated with better performance (table 4).

DSST and PACC showed associations with WMHV, where higher WMHV was associated with poorer performance (table 4).

*APOE* genotype was not associated with any of the cognitive outcomes (accounting for amyloid status and all other factors) (table 4).

There was no evidence of interactions between amyloid status and whole brain volume, or amyloid status and WMHV.

## Discussion

In this large population-based sample of older adults of approximately the same age, we investigated predictors of performance on a range of cognitive measures including the PACC. The key findings are that childhood cognitive ability was strongly associated with all cognitive scores, significant sex differences in cognition were observed, and Aβ positivity and WMHV were associated with lower PACC scores among cognitively normal participants.

Childhood cognitive ability was consistently an important predictor with a notable effect on every cognitive outcome. Our finding that educational attainment and adult SEP were associated with many cognitive outcomes, independent of childhood cognition, is consistent with previous NSHD analyses that have shown that these factors are only moderately correlated and all have direct and indirect influences on cognition across the life course.^38,39^ It is also consistent with evidence that education and occupational attainment may have protective effects on later life cognition.^40^

Women are at greater risk of AD, although this may be partly explained by greater longevity,^41^ and there is evidence of sex differences in the relationships between risk factors and the development of dementia, including *APOE* ε4,^41^ lifestyle factors,^41^ and childhood intelligence,^42^ but the relevance of sex differences to the detection of subtle cognitive decline in preclinical AD is unclear. The effect size of sex on PACC score was large enough to be potentially clinically meaningful (0.4 SD), suggesting that accounting for sex differences on the PACC may be important. As the FNAME test was the component where female participants had the greatest advantage, versions of the PACC that include a different memory test may be less susceptible to sex differences.

The interpretation of the association observed between older age at assessment and lower Matrix Reasoning score is unclear. While scores on this test are known to decline with age, the effect size of our association (−0.17 *z* score units) equates to −0.83 points on the test per year, which is incompatible with the much lower rate of decline across adulthood reported by others.^27,36^ We considered the possibility that our result could be explained by recruitment bias. While participants were invited in a random order, inevitably some participants delayed their visits due to health problems, life circumstances, or being initially undecided about taking part. Therefore participants tested towards the end of the data collection period may have differed in some ways from those seen earlier. However, we did not find any evidence of differences in general health based on measures of self-rated health and overall disease burden, as described elsewhere.^17^

In cognitively normal participants (i.e., excluding those who fulfilled dementia or MCI criteria and those with another neurologic or major psychiatric condition), Aβ positivity was associated with poorer performance on the PACC. Some previous studies have reported similar findings^7^ but others have found no difference between Aβ+ and Aβ− individuals at baseline.^6,8–12^ Statistically significant differences were also observed on several individual tests assessing a range of cognitive domains: memory (Logical Memory Immediate), nonverbal reasoning (Matrix Reasoning), and a global measure of cognitive state (MMSE). These differences were detectable despite there being differences in numbers of participants in the Aβ+ and Aβ− groups—in line with expectations of the proportion of Aβ+ individuals at this age^14^—which reduces statistical power to detect differences between the groups. Our results add to accumulating evidence for subtle cognitive differences associated with Aβ deposition, even at an age when those who are destined to develop dementia are still likely to be many years from symptoms.^13^

In cognitively normal participants with a generally low burden of white matter disease, we also found an independent association between WMHV and PACC score which, to our knowledge, has not been reported before. This suggests that the PACC may be a sensitive, rather than a specific, marker of cerebral pathology—an important consideration for clinical trials.

Controlling for childhood cognitive ability, education and adult SEP enabled detection of a difference in PACC score between Aβ+ and Aβ− participants, whereas the unadjusted group difference was not statistically significant. This may be partially explained by negative confounding effects, whereby one or more factors that predicted higher PACC score also had weak positive associations with Aβ positivity. This was indeed the case for childhood cognitive ability and adult SEP, which were slightly higher in Aβ+ individuals (although differences were not statistically significant). Such differences may well be due to chance but can suppress the association between Aβ and cognition when not adjusted for. Another factor may have been that adjustment for these variables reduced the unexplained residual variance in PACC score, thus increasing the ability to detect smaller differences between the groups. Accounting for IQ may be particularly important in high-functioning individuals.^43^ Combined together, the demographic, life course, and biomarker factors accounted for one third of the variance in PACC score among cognitively normal participants.

Fluid intelligence measures themselves are not usually considered candidates for detecting subtle cognitive decline in preclinical AD, so our finding that Aβ positivity was associated with poorer performance on the Matrix Reasoning test, to a greater degree than the PACC (accounting for childhood cognitive ability), is interesting. It is consistent with evidence that nonverbal IQ declines early in presymptomatic carriers of genetic mutations causing familial AD.^44^ As a high-level test involving multiple domains (including visuoperceptual, working memory, and executive function), Matrix Reasoning is rather different from the tests comprising the PACC, and its potential as a marker of cognitive decline merits further investigation.

The DSST was the single test most sensitive to overall brain health, showing associations with WMHV and whole brain volume in cognitively normal participants, and being the task on which participants with neurologic or major psychiatric conditions were most disadvantaged. Negative effects of WMHV on processing speed are well-established, consistent with subcortical damage.^45^ The DSST may be particularly sensitive to brain pathologies because good performance on this task requires multiple cognitive functions, including visuomotor skills, executive functioning, working memory, and attention, hence people with an impairment in any one of these areas might perform poorly. The fact that the digit–symbol task is timed may also contribute to its sensitivity at detecting small differences in performance.

While some studies have reported effects of *APOE* ε4 on cognition independent of Aβ,^46^ we found that cognition did not differ by *APOE* genotype after accounting for amyloid status.

Few studies have published results of the FNAME test. Our results suggest that the FNAME-12 is sufficiently challenging for 70-year-olds, despite scores on the occupations subscale being somewhat skewed towards the top end. Two previous studies found a sex difference on FNAME, which was reduced in older adults^47^ and attenuated in postmenopausal women.^48^ Here, we found a significant sex difference in 70-year-olds. It has been argued that one potential benefit of the FNAME test is, in contrast to many other memory tests, its reported lack of association with education,^37^ although this has been contradicted in one study.^23^ In the current study, which benefits from prospective collection over the life course, we found that childhood cognitive ability, education, and adult SEP were all significant predictors of FNAME scores.

Two previous studies reported an association between Aβ deposition and FNAME performance, specifically on the FN-N outcome (recall for names).^25,26^ While our results followed this trend, differences between Aβ+ and Aβ− participants did not reach statistical significance in our sample, and the FN-N outcome did not appear more sensitive than FN-O (recall for occupations) or FNAME-total.

This study has a number of major strengths including the prospective collection of clinical and demographic data from birth, the size of the sample, and the very small age range. All participants were from the NSHD, a country-wide cohort of individuals born in the same week, making them more representative than most studies in aging and dementia research, which recruit convenience samples or recruit through memory clinics and may be biased towards those with higher education, higher socioeconomic status, and better cognition.^49^ Insight 46 participants, however, were required to be willing and able to attend a research visit in London, and on average had slightly higher education and SEP than those not in the substudy.^17^ Reflecting the general British postwar population, all NSHD participants are white, so they do not represent the more contemporary ethnic and cultural diversity of the wider population. Within Insight 46, participants with missing neuroimaging data were more likely to be obese and to have mental health problems.^17^ As obesity and depression are associated with increased dementia risk,^50^ this raises the possibility that individuals with AD pathology and associated subtle cognitive decline may be underrepresented in our analyses. Finally, the absence of tau PET imaging precluded investigation of how tau and Aβ pathology may interact to affect cognition.

These data show that childhood cognitive ability, education, and SEP all independently influence cognitive performance at age 70, which has implications both for the interpretation and analysis of cognitive data measured in later life. We provide normative data from a large cohort of Aβ-negative healthy individuals. Our results provide evidence that the PACC can be used to detect subtle cross-sectional differences in cognition associated with Aβ deposition and white matter disease in cognitively normal older adults at an age where dementia prevalence is very low.

## Glossary

Aβ: β-amyloid
AD: Alzheimer disease
CI: confidence interval
DSST: Digit Symbol Substitution Test
FCSRT: Free and Cued Selective Reminding Test
FNAME-12: 12-item Face-Name test
MCI: mild cognitive impairment
MMSE: Mini-Mental State Examination
NSHD: National Survey of Health and Development
PACC: Preclinical Alzheimer Cognitive Composite
SEP: socioeconomic position
SPM: statistical parametric mapping
SUVR: standard uptake volume ratio
TIV: total intracranial volume
WMHV: white matter hyperintensity volume
